# Prediagnostic prescription patterns in pancreatic cancer: a retrospective primary care cohort study

**DOI:** 10.3399/BJGP.2025.0780

**Published:** 2026-06-02

**Authors:** Cecilia Morel, Fiona M Walter, Pawandeep Virpal, Kirsten D Arendse, Garth Funston

**Affiliations:** 1 Centre for Cancer Biomarkers & Biotherapeutics, Barts Cancer Institute, Queen Mary University of London, London, UK; 2 Centre for Cancer Screening, Prevention and Early Diagnosis, Wolfson Institute of Population Health, Queen Mary University of London, London, UK

**Keywords:** diabetes mellitus, early diagnosis, insulin, pancreatic cancer, prescriptions, primary health care

## Abstract

**Background:**

Prescribing patterns in primary care could demonstrate early clinical features of cancer and windows of opportunity for timely investigation.

**Aim:**

To analyse primary care prescription patterns before a pancreatic cancer diagnosis.

**Design and setting:**

Retrospective cohort study using linked primary care and cancer registry data from patients diagnosed with pancreatic cancer in England between 2011 and 2018.

**Method:**

Prescription records registered in the Clinical Practice Research Datalink were analysed in the 5 years prediagnosis. Eight categories of prescriptions that may be used to treat clinical features of pancreatic cancer were included (anti-emetics, anti-reflux medications, insulin, other hypoglycaemic agents, opioids, non-opioid analgesics, neuropathic analgesics, and non-steroidal anti-inflammatories). Poisson regression was used to estimate the inflection points for increased prescribing above baseline.

**Results:**

Among 12 990 patients, 669 287 prescriptions were analysed. Insulin was the least common prescription (6.9% [*n* = 892/12 990] of patients), anti-reflux the most common (52.6% [*n* = 6833/12 990] of patients). Insulin prescribing increased 19 months prediagnosis (95% confidence interval [CI] = 14.2 to 23.8), rising earlier in female patients (25 months; 95% CI = 17.4 to 32.5) than male patients (11 months; 95% CI = 5.8 to 16.2). Prescriptions for other hypoglycaemic agents increased 13 months (95% CI = 7.7 to 18.5), anti-reflux and opioid analgesic prescribing 7 months (95% CI = 5.4 to 8.6 and 4.4 to 9.6, respectively), and anti-emetics and non-opioid analgesics 5 months (95% CI = 2.9 to 7.1 and 3.2 to 6.8, respectively) before diagnosis.

**Conclusion:**

The early increase in insulin prescribing suggests tumour-induced type 3c diabetes, highlighting an opportunity for earlier diagnosis in a small proportion of patients. Opportunities for earlier diagnosis through investigation and referral also exist in patients prescribed anti-emetic, anti-reflux, and analgesic medications in primary care.

## How this fits in

Previous studies across multiple cancer types demonstrate that prescription rates for some medications increase months or years before diagnosis. Assessing whether prescribing changes occur in patients prediagnosis of pancreatic cancer could identify windows for earlier detection. In this study, insulin prescriptions were found to increase 19 months before pancreatic cancer diagnosis, with an earlier rise in female patients compared with male patients (25 months). These findings highlight a potential opportunity for earlier pancreatic diagnosis through prompt investigation of insulin/anti-hyperglycaemic initiation.

## Introduction

In the UK, over 10 000 people are diagnosed with pancreatic cancer each year.^1^ Most (60%) are diagnosed with stage IV disease, which has a 1-year survival of 25%.^2^ Around 70% of people with pancreatic cancer first present to their GP with non-specific symptoms that makes the disease difficult to suspect, and patients frequently present multiple times before referral and diagnosis.^3–5^ Common presenting features of pancreatic cancer include abdominal pain (40%–60%), jaundice (approximately 30%), new-onset diabetes (13%–20%), dyspepsia (approximately 20%), nausea or vomiting (approximately 16%), back pain (approximately 12%), and weight loss (approximately 10%).^6,7^ Around 80% of patients also exhibit impaired glucose tolerance, often directly induced by the tumour — a form classified as type 3c diabetes mellitus (T3cDM).^8,9^ The positive predictive values (PPVs) for these clinical features are, however, <1% in patients aged >60 years, apart from jaundice, which has a reported PPV of 22%.^10^

Earlier cancer diagnosis may be possible by recognising and acting on subtle clinical changes as they first arise in primary care, which could improve patient outcomes. Clinical activities, such as primary care consultations, routine tests, and prescribing, have been noted to increase months to years before diagnosis across several cancer types, demonstrating possible prediagnostic windows for earlier investigations.^11^ Evidence for pancreatic cancer, however, remains limited.^12–16^

This study aimed to analyse prediagnostic primary care prescription patterns among people with pancreatic cancer in England, to identify temporal inflection points (the point at which the prescription rate deviates from the baseline trend) and define possible diagnostic windows where earlier diagnosis might be achieved for some groups. Clinicians must consider disease prevalence and investigate for and treat the most common conditions when patients present with non-specific symptoms, but research is needed to help guide decisions on when to consider more serious, rarer diagnoses such as cancer.

## Method

### Study design

A retrospective cohort study was performed using linked data from Clinical Practice Research Datalink (CPRD) Aurum and the National Cancer Registration and Analysis Service (NCRAS).^17,18^ Patients were included if they had a pancreatic cancer record in NCRAS (*International Classification of Diseases, Tenth Revision* [ICD-10] code C25) between 1 January 2011 and 31 December 2018, were aged ≥18 years at diagnosis, and had no history of an upper gastrointestinal cancer (ICD-10 codes: C15, C16, C23, C24, and C25) before 1 January 2011. Other previous cancer types were not excluded.

### Data sources

CPRD Aurum is an anonymised electronic health record database from patients registered with participating general practices in England.^17^ When a medication is prescribed it is automatically recorded in the patient record, therefore, CPRD prescribing data are highly complete.^19^ CPRD records are linked to NCRAS, which receives data on each primary tumour from multiple sources, has a high degree of accuracy, and complete coverage of all cancer diagnoses in England.^18,20^ Data sources are detailed further in Supplementary Information S1.

### Prescription categories

National Institute for Health and Care Excellence (NICE) guidelines on referral of suspected cancer were used to define possible symptoms/clinical features of pancreatic cancer.^21^ These included back pain, abdominal pain, nausea, vomiting, and new-onset diabetes. Eight medication categories that may be used to treat or manage these features were defined: anti-emetics, anti-reflux agents (protein pump inhibitors, histamine-2 receptor blockers, and antacids), insulin, hypoglycaemic agents, neuropathic analgesics, non-opioid analgesics, non-steroidal anti-inflammatory drugs (NSAIDs), and oral opioids (Box 1). Only oral formulations (tablets, oral sprays, and sublingual) were included, except for injectable hypoglycaemic drugs and topical analgesics. Formulations not typically prescribed in primary care (intravenous, anaesthetic, surgical, and palliative) as well as paediatric formulations were excluded. Lists were prepared and checked by two clinicians (the third and fourth [joint senior] authors). Prescription records from CPRD in the 5 years prediagnosis were included. To avoid capturing medications issued during the final diagnostic phase (during which cancer may be under investigation), prescriptions from the last 2 months prediagnosis (defined as 60.8 days) were excluded.^22^

**Box 1.** Medication categories and examples

Medication category	Subcategories and examples	Corresponding clinical feature(s) of pancreatic cancer
Anti-emetics	Antihistamines (for example, cinnarizine) Phenothiazines (for example, chlorpromazine hydrochloride) 5HT_3_-receptor antagonists (for example, granisetron)	Nausea and vomiting
Anti-reflux agents	PPIs (for example, omeprazole) Histamine-2 receptor blockers (for example, ranitidine) Antacid (for example, alginic acid)	Dyspepsia (heartburn and acid reflux)
Hypoglycaemic agents	GLP-1 receptor agonists (for example, dulaglutide) Alpha-glucosidase inhibitors (for example, acarbose) Biguanides (for example, metformin) DPP-4 inhibitors (for example, alogliptin) Glinides (for example, nateglinide) Sulfonylureas (for example, gliclazide) SGLT2 inhibitors (for example, canagliflozin) Thiazolidinedione (for example, pioglitazone)	Diabetes mellitus (type 2)
Insulin	Insulin	Diabetes mellitus
Neuropathic analgesics	Amitriptyline Pregabalin	Neuropathic pain
Non-opioid analgesics	Paracetamol	Pain
NSAIDs^a^	Naproxen Ibuprofen	Pain (particularly back pain)
Opioid analgesics	Morphine Codeine phosphate	Pain

a Excluding aspirin because this is not commonly used to treat pain in English primary care. DPP-4 = dipeptidyl peptidase-4. GLP-1 = glucagon-like peptide-1. NSAIDs = non-steroidal anti-inflammatory drugs. PPIs = proton pump inhibitors. SGLT2 = sodium-glucose cotransporter-2.

### Clinical and demographic variables

Cancer incidence date, ICD-10 code, and stage (defined using the Tumour Node Metastasis classification system) were sourced from NCRAS. Age was estimated using year of birth from CPRD; as only year was available (to protect anonymity), 1 July was assigned as the date of birth to approximate age at cancer incidence date. Sex was recorded as male or female. Deprivation was recorded using the Index of Multiple Deprivation (IMD) in deciles, where 1 is the most deprived and 10 the least deprived.^23^ Ethnicity was captured from linked data from Hospital Episode Statistics, or, if missing, from CPRD Aurum, and categorised based on CPRD codes into five groups in line with the Office for National Statistics categories: a) White; b) Asian; c) Black; d) mixed; and e) other.^24^

### Analysis

**Figure 1.**
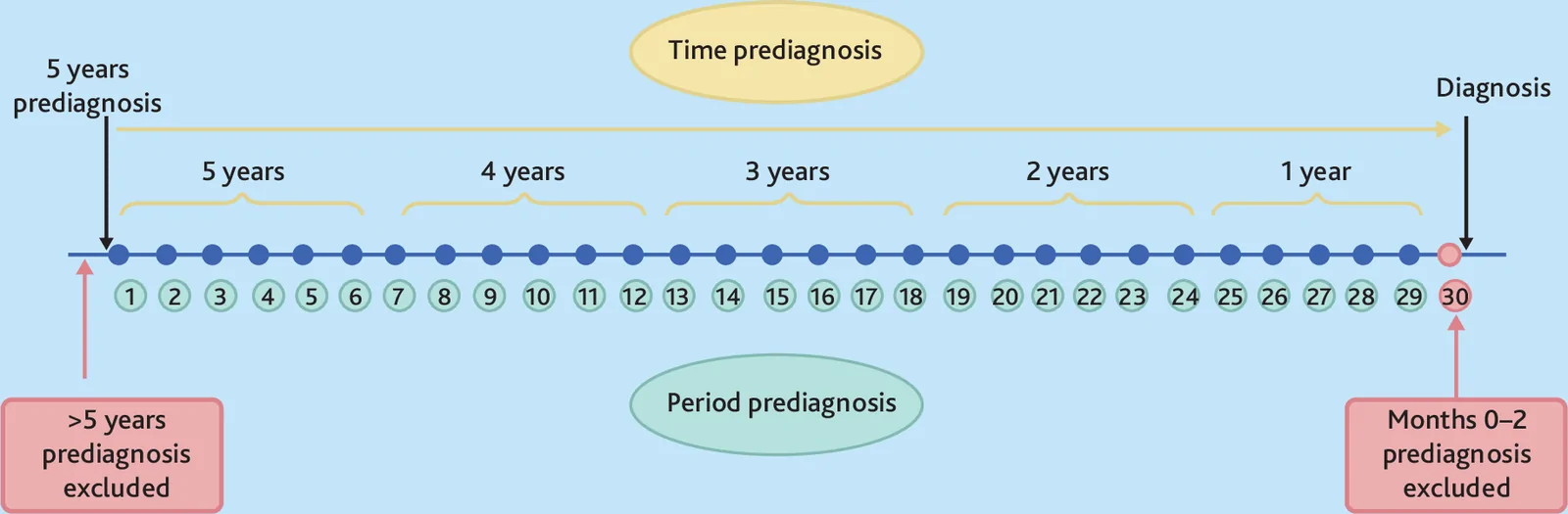
Diagram illustrating how time was divided into bimonthly periods (every 2 months) and years prediagnosis.

Prescription activity was analysed in bimonthly (every 2 months) and annual intervals over the 5 years before diagnosis, divided into 30 bimonthly periods (60.8 days each). Period 30 (2 months prediagnosis) was excluded, leaving 29 periods (Figure 1).

A descriptive analysis was performed examining:

- frequency and rate of prescribing by medication category, stratified by sex; and
- frequency and rate of first-time prescribing by medication category, stratified by sex (where a first-time prescription is defined as no prior prescription ever for that medication, determined by examining records before the 5-year study period).

Multilevel mixed-effects Poisson models, adjusted for age, sex, and deprivation, were used to identify when rates increased from baseline (the inflection point), following joinpoint regression principles — a segmented (or piecewise) regression technique that identifies changes in trends over time by connecting straight-line segments at joinpoints: the point where the rate of change shifts.^25,26^ Although typically applied to continuous data, the concept was adapted to discrete count data by fitting a series of Poisson models to identify potential inflection points.

Methods were partly adopted from Price *et al*.^27^ Each model included a patient-level random intercept to account for within-person clustering and a linear background trend for non-cancer-related prescribing increases.^28^ Periods 2 to 29 were tested as candidate inflection points using a hinge function: values before the point were coded as 0, and values after increased linearly (see Supplementary Table S1 — period 1 was not tested as an inflection point because there were no data preceding it). Separate models were run for each candidate inflection point and included the inflection variable, background trend, random intercept, and covariates (age, sex, and IMD, because these are associated with both pancreatic cancer risk and medication use). Models were run both adjusted and unadjusted for confounders and stratified by sex.

The time period used in the best fitting model (highest log-likelihood) was interpreted as the most likely inflection point. Stability was assessed with non-parametric bootstrapping: 50 bootstrap samples were generated and mean estimates, standard errors, *z*-scores, *P*-values, and 95% confidence intervals (CIs) derived. Inflection points were considered statistically significant if *P*<0.05 and the 95% CIs were between 5 years and 2 months prediagnosis.

Time was converted from periods to months prediagnosis by:^27^

- multiplying by period duration (60.8 days);
- adding half a period (30.4 days) to reflect the middle of the period; and
- dividing by 30.4 to convert days to months.

All analyses, Figures, and Tables were produced in Stata (version 18) and R (version 4.4.1). The reporting of this study followed STROBE guidelines (see Supplementary Information S2).^29^

## Results

In this study, 12 990 patients were included (see Supplementary Figure S1), of which 50.2% (*n* = 6517) were male patients and 49.8% (*n* = 6473) were female patients (Table 1). Median age at diagnosis was 75.7 years among female patients (interquartile range [IQR] 66.7–83.8) and 72.3 years among male patients (IQR 63.7–80.1). Of the 12 990 patients, ethnicity was mainly White (92.7%, *n* = 12 037), the most deprived decile comprised 11.6% (*n* = 1503) of the cohort, decreasing to 8.2% (*n* = 1059) in the least deprived, and 45.1% (*n* = 5853) of patients were diagnosed with stage IV disease.

**Table 1.** Cohort characteristics: patient demographics and clinical characteristics, by sex

Characteristic	Male patients, *n* (%),*N* = 6517	Female patients, *n* (%),*N* = 6473	Total, *n* (%),*N* = 12 990
**Age at diagnosis, years**			
18–39	57 (0.9)	71 (1.1)	128 (1.0)
40–49	278 (4.3)	198 (3.1)	476 (3.7)
50–59	782 (12.0)	569 (8.8)	1351 (10.4)
60–69	1666 (25.6)	1306 (20.2)	2972 (22.9)
70–79	2083 (32.0)	1922 (29.7)	4005 (30.8)
≥80	1651 (25.3)	2407 (37.2)	4058 (31.2)
**Ethnicity**			
White	6037 (92.6)	6000 (92.7)	12 037 (92.7)
Asian	164 (2.5)	150 (2.3)	314 (2.4)
Black	161 (2.5)	153 (2.4)	314 (2.4)
Mixed	25 (0.4)	24 (0.4)	49 (0.4)
Other	58 (0.9)	63 (1.0)	121 (0.9)
Missing	72 (1.1)	83 (1.3)	155 (1.2)
**Deprivation decile**			
1 (most deprived)	739 (11.3)	764 (11.8)	1503 (11.6)
2	718 (11.0)	723 (11.2)	1441 (11.1)
3	709 (10.9)	708 (10.9)	1417 (10.9)
4	731 (11.2)	690 (10.7)	1421 (10.9)
5	607 (9.3)	661 (10.2)	1268 (9.8)
6	642 (9.9)	656 (10.1)	1298 (10.0)
7	664 (10.2)	586 (9.1)	1250 (9.6)
8	606 (9.3)	579 (8.9)	1185 (9.1)
9	567 (8.7)	554 (8.6)	1121 (8.6)
10 (least deprived)	524 (8.0)	535 (8.3)	1059 (8.2)
Missing	10 (0.2)	17 (0.3)	27 (0.2)
**Stage at diagnosis**			
I	347 (5.3)	402 (6.2)	749 (5.8)
II	760 (11.7)	700 (10.8)	1460 (11.2)
III	525 (8.1)	534 (8.2)	1059 (8.2)
IV	3042 (46.7)	2811 (43.4)	5853 (45.1)
Missing	1843 (28.3)	2026 (31.3)	3869 (29.8)

### Prescription rates prediagnosis

In the 5 years prediagnosis, 669 287 prescriptions were issued, with female patients accounting for 53.9% (*n* = 360 999) of these (data not shown). Each female patient, on average, received 55.8 prescriptions and each male patient 47.3 in the 5 years before diagnosis (see Supplementary Table S2).

About half of all 12 990 patients received ≥1 prescription during the 5-year prediagnostic period for anti-reflux medications (52.6%, *n* = 6833), non-opioid analgesics (52.4%, *n* = 6802), opioid analgesics (47.9%, *n* = 6216), and NSAIDs (44.6%, *n* = 5795), whereas insulin (6.9%, *n* = 892) was least frequently prescribed (Table 2).

**Table 2.** Prescription rates: number of patients receiving any prescription and a first-ever prescription in each of the 5 years prediagnosis, by sex

Year before diagnosis	Patients receiving a prescription in each year prediagnosis, *n* (%)			Patients receiving a first-ever prescription in each year prediagnosis, *n* (%)		
	Male patients,*N* = 6517	Female patients,*N* = 6473	Total,*N* = 12 990	Male patients,*N* = 6517	Female patients,*N* = 6473	Total,*N* = 12 990
**Any medication**						
Total period	5954 (91.4)	5885 (90.9)	11 839 (91.1)	726 (11.1)	698 (10.8)	1424 (11.0)
5	4912 (75.4)	5019 (77.5)	9931 (76.5)	153 (2.3)	146 (2.3)	299 (2.3)
4	5027 (77.1)	5136 (79.3)	10 163 (78.2)	141 (2.2)	167 (2.6)	308 (2.4)
3	5115 (78.5)	5219 (80.6)	10 334 (79.6)	160 (2.5)	124 (1.9)	284 (2.2)
2	5205 (79.9)	5274 (81.5)	10 479 (80.7)	142 (2.2)	127 (2.0)	269 (2.1)
1	5336 (81.9)	5322 (82.2)	10 658 (82.0)	130 (2.0)	134 (2.1)	264 (2.0)
**Anti-emetics**						
Total period	869 (13.3)	1570 (24.3)	2439 (18.8)	569 (8.7)	793 (12.3)	1362 (10.5)
5	251 (3.9)	478 (7.4)	729 (5.6)	105 (1.6)	137 (2.1)	242 (1.9)
4	237 (3.6)	500 (7.7)	737 (5.7)	88 (1.4)	131 (2.0)	219 (1.7)
3	252 (3.9)	549 (8.5)	801 (6.2)	80 (1.2)	151 (2.3)	231 (1.8)
2	275 (4.2)	558 (8.6)	833 (6.4)	108 (1.7)	148 (2.3)	256 (2.0)
1	378 (5.8)	688 (10.6)	1066 (8.2)	188 (2.9)	226 (3.5)	414 (3.2)
**Anti-reflux agents**						
Total period	3324 (51.0)	3509 (54.2)	6833 (52.6)	1518 (23.3)	1487 (23.0)	3005 (23.1)
5	1512 (23.2)	1701 (26.3)	3213 (24.7)	242 (3.7)	233 (3.6)	475 (3.7)
4	1639 (25.1)	1899 (29.3)	3538 (27.2)	219 (3.4)	258 (4.0)	477 (3.7)
3	1798 (27.6)	1988 (30.7)	3786 (29.1)	232 (3.6)	232 (3.6)	464 (3.6)
2	1960 (30.1)	2137 (33.0)	4097 (31.5)	264 (4.1)	292 (4.5)	556 (4.3)
1	2540 (39.0)	2654 (41.0)	5194 (40.0)	561 (8.6)	472 (7.3)	1033 (8.0)
**Hypoglycaemic agents**						
Total period	1495 (22.9)	1120 (17.3)	2615 (20.1)	727 (11.2)	572 (8.8)	1299 (10.0)
5	839 (12.9)	597 (9.2)	1436 (11.1)	97 (1.5)	69 (1.1)	166 (1.3)
4	906 (13.9)	667 (10.3)	1573 (12.1)	83 (1.3)	82 (1.3)	165 (1.3)
3	993 (15.2)	727 (11.2)	1720 (13.2)	114 (1.7)	87 (1.3)	201 (1.5)
2	1104 (16.9)	829 (12.8)	1933 (14.9)	143 (2.2)	118 (1.8)	261 (2.0)
1	1354 (20.8)	1003 (15.5)	2357 (18.1)	290 (4.4)	216 (3.3)	506 (3.9)
**Insulin**						
Total period	528 (8.1)	364 (5.6)	892 (6.9)	289 (4.4)	231 (3.6)	520 (4.0)
5	253 (3.9)	153 (2.4)	406 (3.1)	24 (0.4)	26 (0.4)	50 (0.4)
4	280 (4.3)	167 (2.6)	447 (3.4)	31 (0.5)	20 (0.3)	51 (0.4)
3	319 (4.9)	187 (2.9)	506 (3.9)	43 (0.7)	32 (0.5)	75 (0.6)
2	358 (5.5)	234 (3.6)	592 (4.6)	52 (0.8)	47 (0.7)	99 (0.8)
1	491 (7.5)	332 (5.1)	823 (6.3)	139 (2.1)	106 (1.6)	245 (1.9)
**Neuropathic analgesics**						
Total period	978 (15.0)	1378 (21.3)	2356 (18.1)	566 (8.7)	678 (10.5)	1244 (9.6)
5	368 (5.6)	600 (9.3)	968 (7.5)	87 (1.3)	149 (2.3)	236 (1.8)
4	408 (6.3)	656 (10.1)	1064 (8.2)	95 (1.5)	124 (1.9)	219 (1.7)
3	459 (7.0)	695 (10.7)	1154 (8.9)	116 (1.8)	118 (1.8)	234 (1.8)
2	505 (7.7)	754 (11.6)	1259 (9.7)	112 (1.7)	147 (2.3)	259 (2.0)
1	546 (8.4)	769 (11.9)	1315 (10.1)	156 (2.4)	140 (2.2)	296 (2.3)
**Non-opioid analgesics**						
Total period	3117 (47.8)	3685 (56.9)	6802 (52.4)	977 (15.0)	940 (14.5)	1917 (14.8)
5	1527 (23.4)	2061 (31.8)	3588 (27.6)	204 (3.1)	197 (3.0)	401 (3.1)
4	1597 (24.5)	2185 (33.8)	3782 (29.1)	165 (2.5)	185 (2.9)	350 (2.7)
3	1679 (25.8)	2175 (33.6)	3854 (29.7)	184 (2.8)	165 (2.5)	349 (2.7)
2	1723 (26.4)	2257 (34.9)	3980 (30.6)	184 (2.8)	174 (2.7)	358 (2.8)
1	1875 (28.8)	2381 (36.8)	4256 (32.8)	240 (3.7)	219 (3.4)	459 (3.5)
**NSAIDs**						
Total period	2748 (42.2)	3047 (47.1)	5795 (44.6)	737 (11.3)	775 (12.0)	1512 (11.6)
5	1209 (18.6)	1304 (20.1)	2513 (19.3)	165 (2.5)	161 (2.5)	326 (2.5)
4	1191 (18.3)	1358 (21.0)	2549 (19.6)	161 (2.5)	175 (2.7)	336 (2.6)
3	1181 (18.1)	1312 (20.3)	2493 (19.2)	145 (2.2)	145 (2.2)	290 (2.2)
2	1141 (17.5)	1304 (20.1)	2445 (18.8)	136 (2.1)	162 (2.5)	298 (2.3)
1	1070 (16.4)	1260 (19.5)	2330 (17.9)	130 (2.0)	132 (2.0)	262 (2.0)
**Opioid analgesics**						
Total period	2883 (44.2)	3333 (51.5)	6216 (47.9)	950 (14.6)	890 (13.7)	1840 (14.2)
5	1282 (19.7)	1663 (25.7)	2945 (22.7)	180 (2.8)	166 (2.6)	346 (2.7)
4	1333 (20.5)	1763 (27.2)	3096 (23.8)	192 (2.9)	185 (2.9)	377 (2.9)
3	1365 (20.9)	1770 (27.3)	3135 (24.1)	171 (2.6)	153 (2.4)	324 (2.5)
2	1370 (21.0)	1807 (27.9)	3177 (24.5)	167 (2.6)	162 (2.5)	329 (2.5)
1	1589 (24.4)	1933 (29.9)	3522 (27.1)	240 (3.7)	224 (3.5)	464 (3.6)

NSAIDs = non-steroidal anti-inflammatory drugs.

Overall, 12 699 first-ever prescriptions were issued (data not shown). Anti-reflux drugs were most common (23.3% [*n* = 1518/6517] of male patients and 23.0% [*n* = 1487/6473] of female patients), followed by non-opioid analgesics, opioid analgesics, and NSAIDs (Table 2). Sex differences were small, the largest being a 3.6% higher rate of first-time anti-emetic prescribing in female patients.

Prescription rates rose gradually prediagnosis for all medication categories except NSAIDs, which declined slightly (Table 2 and Supplementary Figure S2a). The largest increases were for anti-reflux drugs (from 24.7% [*n* = 3213] at 5 years to 40.0% [*n* = 5194] at 1 year prediagnosis), non-opioid analgesics (from 27.6% [*n* = 3588] to 32.8% [*n* = 4256]), and opioid analgesics (from 22.7% [*n* = 2945] to 27.1% [*n* = 3522]) (Table 2), with marked rises 5–7 months prediagnosis. Unlike overall rates, first-time prescription rates remained relatively stable, not increasing until a sharp rise 5–7 months prediagnosis (except for NSAIDs) (see Supplementary Figure S2b). First-time insulin prescribing more than doubled from 0.8% (*n* = 99) in the 2 years prediagnosis to 1.9% (*n* = 245) in the year before diagnosis (Table 2).

### Inflection point estimates

**Figure 2.**
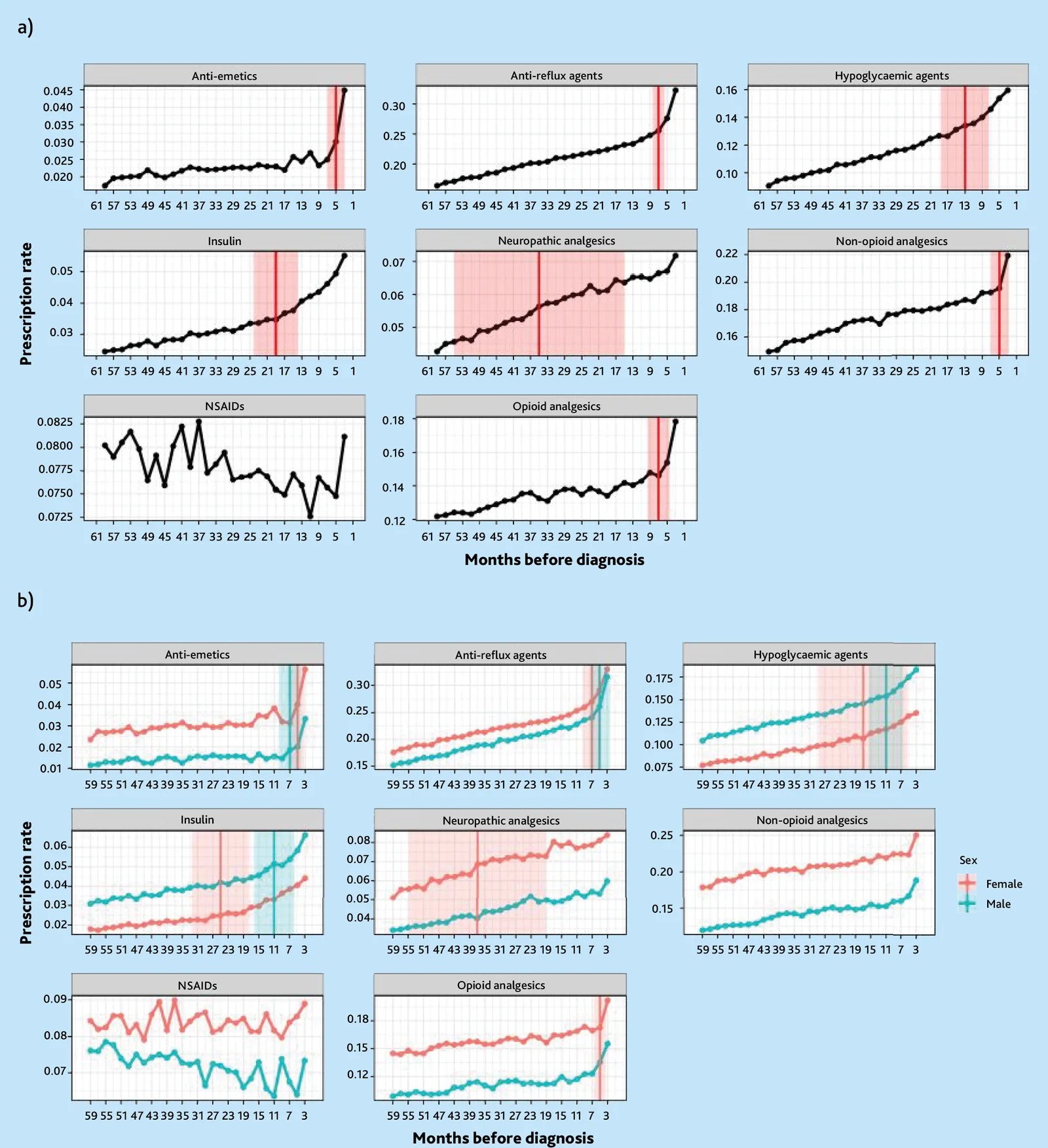
Prescription rates in the 5 years before pancreatic cancer diagnosis, with inflection points marked by vertical lines and corresponding 95% confidence intervals shown as shaded areas. Each data point represents a 2-month interval, plotted according to the midpoint of the interval (for example, the point at 3 months represents prescriptions issued between 2 and 4 months before diagnosis). a) All patients combined. b) Stratified by sex. NSAIDs = non-steroidal anti-inflammatory drugs.

Inflection points were identified for all medication categories except NSAIDs, for which the 95% CI extended 27 months into the diagnostic period (5 months prediagnosis, 95% CI = –27.4 to 35.6) (Figure 2). Neuropathic analgesics increased earliest but CIs were very wide (35 months; 95% CI = 15.0 to 55.0), followed by insulin (19 months; 95% CI = 14.2 to 23.8) and hypoglycaemic agents (13 months; 95% CI = 7.7 to 18.5). Anti-reflux and opioid analgesic prescribing rose above baseline at 7 months (95% CI = 5.4 to 8.6 and 4.4 to 9.6, respectively), and non-opioid analgesics and anti-emetics at 5 months (95% CI = 3.2 to 6.8 and 2.9 to 7.1, respectively) prediagnosis. Unadjusted estimates are provided in Supplementary Table S3.

When stratified by sex, inflection points were noted for female patients for all prescriptions except NSAIDs and non-opioid analgesics, whereas for male patients, inflection points were only observed for anti-emetics, anti-reflux drugs, hypoglycaemic agents, and insulin (for all others, 95% CIs extended into the 2 months prediagnosis). Insulin prescriptions increased earlier in female patients (25 months, 95% CI = 17.4 to 32.5) compared with male patients (11 months, 95% CI = 5.8 to 16.2), with independent CIs (although these were broad for female patients). Although there were differences in inflection points between male patients and female patients for other medication categories, CIs overlapped (Figure 2).

## Discussion

### Summary

Prescription rates increased above baseline for seven medication categories in the lead-up to pancreatic cancer diagnosis. Insulin prescribing rates rose 19 months prediagnosis — earlier in female patients (25 months) than male patients (11 months) (but with wide CIs for female patients and only a small proportion of the cohort [approximately 2%] initiating treatment ≥1 year prediagnosis). Hypoglycaemic agent prescriptions increased at 13 months prediagnosis (approximately 6% initiating treatment ≥1 year prediagnosis), whereas later increases were observed for anti-reflux drugs (7 months), opioid analgesics (7 months), anti-emetics (5 months), and non-opioid analgesics (5 months), with no differences by sex. Neuropathic analgesic prescribing increased earliest (35 months) but CIs were extremely broad. These findings suggest that prescribing for conditions such as diabetes, dyspepsia, nausea and vomiting, and pain could indicate underlying pancreatic cancer or related diseases. Despite some uncertainty around inflection points, these findings highlight possible extended diagnostic windows in which investigation of patients presenting with some clinical features, or being prescribed specific medications, could contribute to earlier cancer diagnosis.

### Strengths and limitations

This study used a large, nationally representative primary care dataset linked to cancer registry data, providing the gold standard for cancer case identification.^17,18^ Additionally, prescriptions were automatically recorded in patient records, minimising the risk of underrecording (which often occurs for manually coded symptoms).^30^ Unlike previous studies examining changes in medication use before pancreatic cancer diagnosis, this study estimated inflection points using a maximum-likelihood approach, which uses an individual’s own prescribing history to determine the baseline rate and provides CIs around the inflection point.^27^

However, this population-level analysis captured aggregated prescribing trends rather than individual predictive values. It is not possible using these methods to determine the number of prescriptions that should trigger further investigation. Further, the proportion of patients driving these changes could not be examined, so a minority of patients with atypical cases could theoretically explain the patterns.^31^ Formal diagnostic assessments (beyond log-likelihood metrics) were also not undertaken to evaluate the fit of the Poisson models — prescription count data often exhibit substantial variability, therefore overdispersion may remain. Additionally, the evaluation of multiple medication categories, stratified by sex, within the same dataset introduces a risk of type I error. Although the current study accounted for age, sex, and deprivation, residual confounding from factors such as alcohol use, body mass index, and comorbidities may remain.

Prescriptions for other symptoms that may be indicative of pancreatic cancer, such as diarrhoea and constipation,^21^ were also not analysed — these results do not therefore reflect the full prescribing profile of pancreatic cancer. Additionally, as CPRD captures only primary care prescriptions, it was not possible to account for prescriptions issued in secondary care, the private health sector, or over-the-counter use. Furthermore, it was not possible to determine indications for the medication, nor whether the prescription was redeemed or the medication taken. Nonetheless, analysing changes in rates rather than absolute levels minimises the influence of unrelated chronic prescribing.^32^

### Comparison with existing literature

In the UK, Denmark, and the Netherlands prescription rates rise months before cancer diagnoses, with figures of 5–12 months for lung,^33,34^ 12–18 months for colorectal,^35,36^ 9 months for bladder and renal,^28^ 7 months for Hodgkin lymphoma,^37^ and 6 months for all cancers combined, with proton pump inhibitors and analgesics, particularly opioids, showing the greatest increases.^38^ Diagnostic windows across cancer types and primary care activities typically range from 5 to 24 months.^22,28,32,33,35,37,39–43^ For pancreatic cancer, Danish studies found increases in GP consultations, hospital admissions and visits, and glucose testing 7–12 months before diagnosis, and rises in imaging and abdominal investigations 5–6 months before diagnosis.^12,13^

Evidence on prescription use before diagnosis of pancreatic cancer mainly concern insulin: recent use (<5 years) is consistently linked to increased risk of diagnosis,^14–16,44,45^ and one study also reported a similar association for anti-reflux medications.^15^ These studies used logistic regression to estimate odds or hazard ratios and were often limited by small sample size; the current study applied Poisson regression with a maximum-likelihood method to identify the point at which prescribing rates deviated from baseline, using a large cohort.

Consistent with previous studies, prescribing for insulin and hypoglycaemic agents increased before diagnosis, likely because of tumour-induced diabetes (T3cDM), characterised by rapid glycaemic deterioration and weight loss.^46–49^ The rise in first-time insulin prescriptions 17 months prediagnosis (see Supplementary Figure S2) supports evidence that new-onset diabetes precedes pancreatic cancer by 2–3 years.^16,45,50,51^ Experimental data show that tumour-secreted factors impair β-cell function and glucose metabolism, and that glycaemic control improves after tumour resection, suggesting a tumour-driven mechanism.^52–55^ Thus, the earlier rise in insulin prescribing relative to hypoglycaemic agents suggests that in many individuals with pancreatic cancer, diabetes is acute and severe, consistent with β-cell decompensation rather than the gradual development of insulin resistance seen in type 2 diabetes.

Sex differences may reflect biological or behavioural factors. Rasmussen *et al*’s study reported earlier glucose testing in male patients compared with female patients with pancreatic cancer,^12^ although methodological differences or differences in prescribing practices between countries may explain the discrepancy.

### Implications for research and practice

The early rise in insulin prescribing, particularly among female patients, likely reflects T3cDM secondary to pancreatic cancer. Although distinguishing T3cDM from type 2 diabetes is challenging, it may present with persistently elevated glycosylated haemoglobin despite treatment, greater prediagnostic weight loss, and later age at onset.^47,48,56^ These findings suggest that new insulin initiation in older adults should raise suspicion for pancreatic cancer, consistent with NICE guidance recommending urgent computed tomography imaging for patients aged >60 years with unexplained weight loss and new-onset diabetes.^21^ This study, together with the UK Early Detection Initiative,^57^ further supports biomarker-based screening for pancreatic cancer in new-onset diabetes cohorts. However, considering only 14% of patients started insulin or hypoglycaemic treatment during the 5-year study period, this would benefit a minority of patients.

Inflection points for anti-emetic, anti-reflux, and analgesic medications occur close to diagnosis and are likely late markers of disease rather than early signals of disease presentation. Additionally, although the 2 months before diagnosis was excluded, some outlier patients may have longer intervals between initial clinical suspicion and confirmed diagnosis. Therefore, later inflection points may be of limited clinical value for early detection and should be interpreted with caution.

Further research should clarify sex differences, examine first-time insulin prescribing in patients without prior hypoglycaemic use (to identify true new-onset insulin-dependent diabetes), and assess concurrent prescribing across medication categories as an indicator of condition severity. Absolute increases in anti-diabetic prescribing are modest (approximately 3% for insulin and approximately 7% for hypoglycaemic agents over 5 years), so further research is needed to determine whether these changes would be detectable in routine practice. These findings also require validation in more diverse international cohorts and cannot be directly applied to primary care without further studies assessing the PPVs of specific prescriptions. The PPVs of the individual clinical features these medications are prescribed for are below the 3% NICE referral threshold for referral of suspected cancer;^51^ however, combining features and prescribing patterns within prediction models could improve risk stratification and support stratification of referral thresholds by specific groups, for example, age group. Recent models, such as ENDPAC (Enriching New-Onset Diabetes for Pancreatic Cancer),^58,59^ among others,^60–63^ can accurately identify high-risk individuals, with ENDPAC being assessed for use in the UK.^64^ Current risk assessment tools in UK primary care are for multiple cancers and do not integrate prescription data.^65^ This study suggests a window exists in which pancreatic cancer-specific tools could be implemented for earlier detection. Integration of prescribing data and biomarkers distinguishing T3cDM from type 2 diabetes (for example, adiponectin and interleukin-1 receptor antagonist)^46^ may improve risk stratification and enable some individuals with pancreatic cancer to be diagnosed at an earlier stage; however, further studies are required to examine this.

Finally, many anti-emetic, anti-reflux, and analgesic medicines are obtained via community pharmacy prescribing or over the counter, meaning primary care data likely underestimate community symptom management. This is increasingly relevant as pharmacy-based clinical care expands in England: from 2026, all newly qualified pharmacists will register as independent prescribers,^66^ and NHS policy supports pharmacists in arranging tests for possible cancer symptoms.^67^ Community pharmacies are therefore becoming key contact points for people with non-specific symptoms, including those linked to pancreatic cancer. Many patients self-manage suspected cancer symptoms with over-the-counter products,^68^ and approaches such as the Cancer Loyalty Card Study highlight opportunities to identify symptom-related purchasing beyond traditional healthcare data.^69^ This shift towards community-based care may enable earlier identification and referral, although further research in this setting is needed.

## Data

This study is based in part on data from the CPRD obtained under license from the UK Medicines and Healthcare products Regulatory Agency. This study made use of data from Health Episode Statistics and National Cancer Registration and Analysis System copyright © 2026, re-used with the permission of the Health and Social Care Information Centre. All rights reserved. The data are provided by patients and collected by the NHS as part of their care and support. The interpretation and conclusions contained in this study are those of the authors alone. All scripts used to analyse the data will be made available, as well as the results. All code lists will be made available on Queen Mary Research Online (https://qmro.qmul.ac.uk/xmlui).
